# RNA Interference in the Age of CRISPR: Will CRISPR Interfere with RNAi?

**DOI:** 10.3390/ijms17030291

**Published:** 2016-02-26

**Authors:** Unnikrishnan Unniyampurath, Rajendra Pilankatta, Manoj N. Krishnan

**Affiliations:** 1 Program on Emerging Infectious Diseases, Duke-NUS Medical School, 8 College Road, Singapore 169857, Singapore; unni@duke-nus.edu.sg; 2 Department of Biochemistry and Molecular Biology, School of Biological Sciences, Central University of Kerala, Nileshwar 671328, India; praj74@gmail.com

**Keywords:** RNAi interference, RNAi, CRISPR, Cas9, genome engineering, reverse genetic screens

## Abstract

The recent emergence of multiple technologies for modifying gene structure has revolutionized mammalian biomedical research and enhanced the promises of gene therapy. Over the past decade, RNA interference (RNAi) based technologies widely dominated various research applications involving experimental modulation of gene expression at the post-transcriptional level. Recently, a new gene editing technology, Clustered Regularly Interspaced Short Palindromic Repeats (CRISPR) and the CRISPR-associated protein 9 (Cas9) (CRISPR/Cas9) system, has received unprecedented acceptance in the scientific community for a variety of genetic applications. Unlike RNAi, the CRISPR/Cas9 system is bestowed with the ability to introduce heritable precision insertions and deletions in the eukaryotic genome. The combination of popularity and superior capabilities of CRISPR/Cas9 system raises the possibility that this technology may occupy the roles currently served by RNAi and may even make RNAi obsolete. We performed a comparative analysis of the technical aspects and applications of the CRISPR/Cas9 system and RNAi in mammalian systems, with the purpose of charting out a predictive picture on whether the CRISPR/Cas9 system will eclipse the existence and future of RNAi. The conclusion drawn from this analysis is that RNAi will still occupy specific domains of biomedical research and clinical applications, under the current state of development of these technologies. However, further improvements in CRISPR/Cas9 based technology may ultimately enable it to dominate RNAi in the long term.

## 1. Introduction

**Figure 1:**
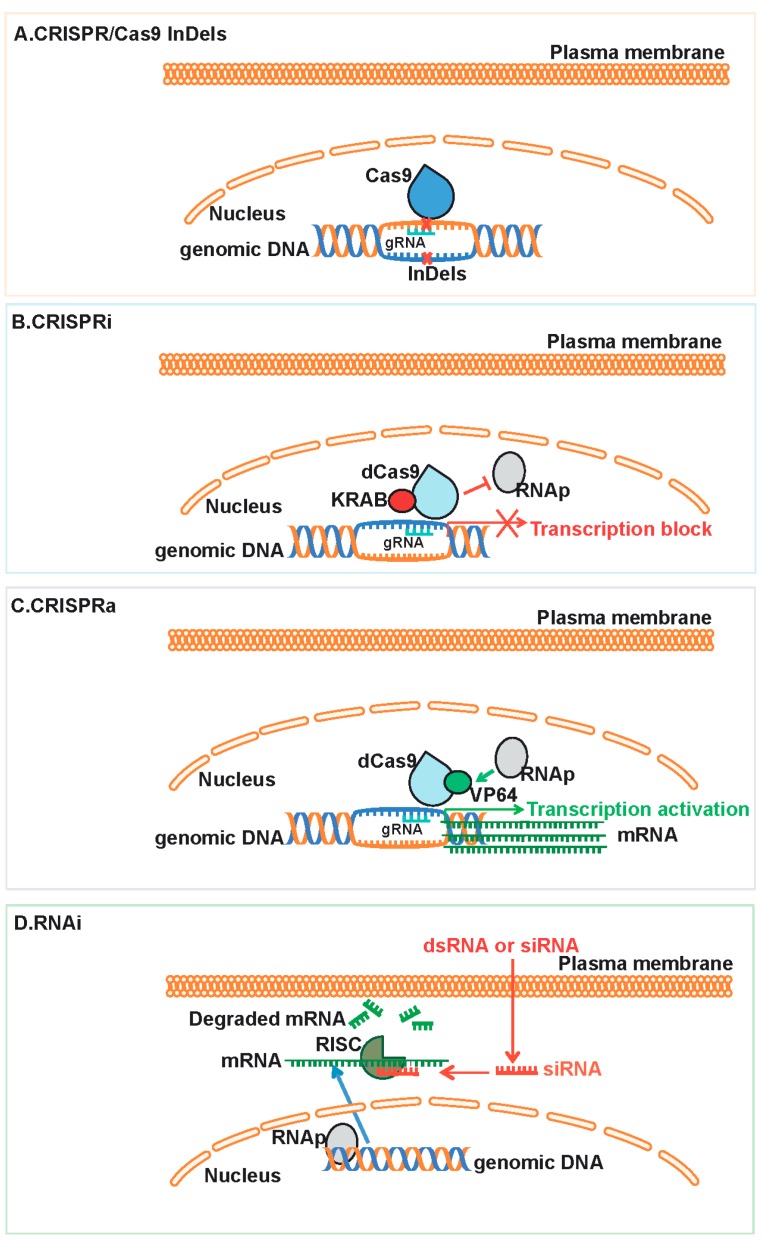
Schematic representation of CRISPR/Cas9 systems and RNAi. The general functioning of CRISPR/Cas9 systems and RNAi are displayed. (**A**) CRISPR/Cas9 induces insertions and deletions (InDel) when targeted to a specific genomic site with the help of appropriate guide RNA (gRNA); (**B**) CRISPR interference (CRISPRi) down-regulates gene transcription; (**C**) CRISPR activation (CRISPRa) up-regulates gene transcription; (**D**) RNA interference (RNAi) down regulates gene transcription by acting post-transcriptionally. dCas, catalytic mutant Cas9; siRNA, small interfering RNA; dsRNA, double stranded RNA; RNAp, RNA polymerase; RISC, RNA induced silencing complex.

The RNA interference (RNAi), a powerful transient gene-expression repression approach discovered over a decade ago, has brought unprecedented applications in the research on gene-phenotype relationship in mammalian systems [1,2]. Parallel to the development of RNAi, several other techniques for stable genetic modifications have also evolved, collectively called gene editing techniques [3]. One such gene editing approach is based on the Clustered Regularly Interspaced Short Palindromic Repeats (CRISPR)/CRISPR-associated protein 9 (Cas9) systems present in prokaryotes for nucleic acid editing. The application of CRISPR/Cas9 for general DNA editing as well as for mammalian gene editing was established in the 2012–2013 period, and, in just three years, this technique has revolutionized the entire gene editing field [4,5,6]. While RNAi dominated the mammalian gene expression manipulation field for the past 15 years, the recent rapid growth of CRISPR/Cas9 raises the question whether RNAi will become a tool of the past or not. This review provides a concise account on the origins and mechanisms of RNAi and CRISPR/Cas9, along with a detailed analysis on whether either one of these two techniques will outweigh the other in terms of their applications. The description below pertains only to the applications of CRISPR/Cas9 and RNAi in the mammalian systems. A schematic showing currently available options of gene editing using CRISPR/Cas9 systems and RNAi are given in Figure 1.

## 2. CRISPR: Prokaryotic Adaptive Immune Response System to Powerful Eukaryotic Gene Editing Tool

The CRISPR/Cas systems is a naturally occurring prokaryotic immune defense strategy that has been recently adapted for eukaryotic genome engineering. The potential of the CRISPR/Cas system to edit eukaryotic genomes offers a wide variety of therapeutic, biomedical, agricultural and research applications.

### 2.1. CRISPR: A Prokaryotic Adaptive Immune Response System

The CRISPR/Cas system confers adaptive immune protection to prokaryotes such as bacteria and archaea against non-self DNA based invasions (e.g., viruses, plasmids) in a sequence-specific manner [7,8]. The CRISPR-Cas loci are present in both bacteria and archaea (48% and 84% respectively) [9]. Although the initial observation on the presence of clustered repeats of genetic elements from the genome of the bacterium *Escherichia coli* was made in 1987, the term CRISPR was coined more than a decade later [10,11]. Subsequently, the CRISPR system was identified to target invading DNA elements [12,13,14,15].

### 2.2. CRISPR: Biochemical Architecture

The CRISPR/Cas systems that are present in diverse organisms essentially have a comparable core genetic organization [16,17,18]. They have several DNA repeat elements interspersed with short “spacer” sequences derived from foreign DNA, and multiple *Cas* genes, some of which are nucleases [19]. The spacer sequence constitutes a code for the respective foreign genetic element, which will be used by the host prokaryote to quickly identify any homologous sequence subsequently entering the host cell.

The CRISPR/Cas system functions in three steps [20,21]. It generates immunity by first genetically tethering invading phage and plasmid DNA segments (spacers) into CRISPR loci (called the spacer acquisition step) [22,23,24]. Subsequently, the host prokaryotic organism will transcribe and process CRISPR loci to generate mature CRISPR RNA (crRNA) containing both CRISPR repeat elements and the integrated spacer genetic segment of the foreign DNA corresponding to the previous non-self genetic invasion (called crRNA maturation step) [12,25,26,27]. Finally, the crRNA will detect homologous DNA sequences by complementary base pairing when newly introduced, such as during infection with another virus having the same sequence. Once crRNA detects foreign DNA, the invading non-self genetic material will be clipped through a complex nucleic acid processing biochemical machinery, yielding protection against foreign DNA (called the target interference step) [7,22,28,29].

The Cas proteins are involved in all three major steps of CRISPR/Cas system functioning. Among the ten superfamilies of Cas proteins (Cas1-Cas10) [30], Cas1 and 2 are primarily involved in spacer acquisition [31,32], Cas6 and 5d mediate crRNA maturation [16,25,33], and several other remaining Cas proteins are part of the interference complex.

The CRISPR/Cas systems have been classified into three major types based on the conservation and composition of the Cas genes [30,34]. The type II is present exclusively in bacteria, while types I and III systems are present in both bacteria and archaea. All three types of CRISPR/Cas systems are capable of editing DNA. The two major differences between the three types of CRISPR/Cas systems are: (i) target: Type II and III systems have DNA and RNA targeting subclasses. The type I system targets only DNA; and (ii) subunit composition. The type II system has two different RNA subunits in complex with a single Cas9 protein. Type I and III systems have multiple Cas proteins in complex with a single RNA.

### 2.3. CRISPR/Cas9, CRISPRi and CRISPRa: Tools for Eukaryotic Gene Editing

Recently, CRISPR/Cas system has been adapted for RNA-directed DNA editing of eukaryotic genomes including mammalian systems. The original identification that the CRISPR/Cas system can edit DNA subsequently catalyzed the thinking that Cas proteins with nuclease function could be potentially adapted for genome engineering of higher eukaryotes [5]. Validating this line of thinking, it was eventually established in 2013 that the CRISPR/Cas type II system, specifically the CRISPR/Cas9 system, could be engineered to edit mammalian genomes [4,6].

Among the three types of CRISPR/Cas systems, only type II has been widely adapted for eukaryotic gene editing [4,6,20]. A major reason for this is the simplicity of the type II system, in which only the Cas9 was needed for both recognizing target DNA regions and creating the gene modification using an appropriate crRNA. The type II CRISPR/Cas system required only the tracrRNA cofactor, the crRNA and Cas9 to induce gene editing. All other CRISPR/Cas types needed multiple components, with still poorly understood regulatory mechanisms [8,20,34].

The basal CRISPR/Cas9 machinery was originally adapted for introducing stable insertions and deletions (InDels) in the mammalian genome. However, there could be several applications that will only require transient repression or activation of gene expression. Subsequently, Cas9 was engineered to also perform transient repression and activation of mammalian genes. This was based on the principle that gRNA can target Cas9 to any destination segment of the genome. The ability of gRNA to target Cas9 to any destination segment of the genome could be exploited to alter gene expression levels if repressor or activator functionalities are conjoined to this machinery. On this line, it was later determined that fusion of transcription repression (e.g., Krüppel associated box KRAB) or activation (e.g., VP64) domains to catalytically inactive Cas9 molecule (called dCas9) could result in respectively transcription repression (called CRISPR interference, CRISPRi) or activation (called CRISPR activation, CRISPRa) of a gene targeted using appropriate gRNA [35,36,37]. Essentially, the gRNA could guide the dCas9 to the promoter regions of the intended target gene, and the repression or activation domains could modify the transcription. In many ways, CRISPRi works analogous to RNAi, although the underlying mechanisms are dissimilar. Both CRISPRi and RNAi are transient gene expression repression approaches. While CRISPRi transiently represses gene expression at the level of DNA, RNAi employs post-transcriptional mechanisms. The duration of transcript repression exerted by both of these approaches directly corresponds to the time the targeting components (dCas9-fusion domain + gRNA for CRISPRi, and siRNA or shRNA for RNAi) are present in the cells at the adequate doses.

The mammalian cells can respond to CRISPR/Cas9 mediated DNA breaks by adopting any one of the two repair mechanisms. The host cell will fix the DNA damage caused by CRISPR/Cas9 through either non-homologous end joining method (NHEJ) or homology directed repair (HDR) [38,39]. In NHEJ, the host cellular DNA repair systems will tether the DNA double strand break by random insertion or deletion of short stretches of oligonucleotide bases. This could potentially lead to the disruption of the codon-reading frame, resulting in erroneous transcripts and ablation of gene expression. In HDR, introduction of a segment of DNA with regions having homology to the sequences flanking both sides of the DNA double strand break will lead to the repair by host machinery through the incorporation of the extra segment of the DNA fragment. Both these types of host DNA repair response mechanisms to CRISPR/Cas9 mediated DNA double strand break offers various types of applications for CRISPR/Cas9 in mammalian gene editing. NHEJ can lead to ablation of gene mutations, and hence can be used to create loss of function effects. HDR can be used for introducing specific point mutations, or introducing DNA segments of varying length.

## 3. RNAi: Evolutionarily Conserved Eukaryotic Post-Transcriptional Gene Expression Regulation Mechanism

### 3.1. RNAi: Eukaryotic Post-Transcriptional Gene Expression Regulation Mechanism

As a mature field, RNAi has been well reviewed elsewhere [40,41,42]. Here, we will provide a very brief overview of the RNAi principles. RNA interference is a double stranded RNA mediated homology based mechanism evolved to post-transcriptionally regulate eukaryotic gene expression and to serve in host immunity. The discovery of the phenomenon of RNAi happened over a long stretch of time. Phenotypic effects of RNAi, and RNA induced homologous endogenous mRNA suppression were initially observed in plants while experimenting for flower color alterations [43]. Plant scientists refereed to this phenomenon as cosuppression. The RNAi, as it stands today, was first observed in *Caenorhabditis elegans*, through the discovery that gene expression could be modified using double stranded RNA [1]. The core component of RNAi is the formation of small interfering RNAs (siRNAs) with regions homologous to the intended cellular RNA targets [2]. The siRNAs are generated endogenously by processing long double stranded RNAs in the cytoplasm, leading to the formation of 21-nt double stranded RNA with a 3’ overhang of two nucleotides, and a 5’ phosphate group. This ~21-mer will finally pair with the intended homologous sequence containing target mRNAs, and induce their degradation through a biochemical complex called RNA induced silencing complex (RISC). RNAi could also be induced by direct exogenous supply of short double stranded RNAs that are ~21 nucleotides in length, called small interfering RNAs (siRNA). Alternatively, RNAi can also be introduced into cells using plasmid delivery of short hairpin RNAs (shRNAs), which will be processed similarly to generate siRNAs inside the cytoplasm [44]. In mammalian systems, RNAi is best achieved by the delivery of small double stranded RNAs, because long double stranded RNAs have been shown to induce potent toxic interferon response. As most of the applications of RNAi are achieved through exogenous supply of chemically synthesized siRNAs, the rest of this review will discuss on the mechanism involved in siRNA based induction of RNAi to repress eukaryotic genes [45].

### 3.2. RNAi: Biochemical Mechinery

The core operation of the RNAi involves two steps. In Step 1, the double stranded RNA will be processed in the cytoplasm by an RNAse III type endoribonuclease called Dicer to generate ~21 nucleotide long siRNAs [46]. Subsequently, siRNA bound Dicer will transfer small dsRNA to Argonaute, with the help of double stranded RNA binding proteins (dsRNABP) [47]. Subsequently, in Step 2, Argonaute will bind one strand of the duplex (guide strand), while the other strand will be displaced [48,49,50]. This whole protein-RNA complex is called RISC. The fully charged RISC will survey for long RNAs that can pair with the single stranded guide RNA bound to the Argonaute. Upon engagement with a long homologous RNA target that pairs with the guide RNA, Argonaute will be activated and degrade the target RNA though its RNAse-H like activity.

## 4. Comparison of the Applications of CRISPR/Cas9 and RNAi in the Mammalian Biomedical Field: Will CRISPR Delete RNAi or Only Moderately Repress?

Recently the CRISPR/Cas9 system has received wide acceptance as a robust and versatile tool for gene editing, primarily because of both its simplicity and wider capabilities. Until now, RNAi has been the major dominating genetic tool for several applications in mammalian biomedical field requiring gene expression modification. The rapid growth and use of CRISPR/Cas9 in mammalian systems warrants an analysis on how it will impact on the future of the uses and applications of RNAi in biomedical research. Will CRISPR/Cas9 replace RNAi, or, will these two applications have unique space in the biomedical research? On the mechanistic side, these two technologies are fundamentally different. While the primary use of the CRISPR/Cas9 system is to induce gene editing, RNAi is a post-transcriptional gene expression modifier process. This fundamental difference seems to indicate that these two technologies may occupy unique non-overlapping spaces in biomedical research. However, a closer analysis of the modifications and applications of these two technologies reveals that CRISPR/Cas9 and RNAi also occupy several overlapping domains of biomedical research, raising the possibility that one may selectively dominate over the other.

In general, the applications of both CRISPR/cas9 and RNAi can be classified into two major domains: (i) research tool applications; and (ii) clinical applications. We have provided below a comparative analysis of the scope of CRISPR/Cas9 and RNAi in the research tool field and clinical applications. In addition, we have also discussed some of the “technical considerations” associated with each of these technologies that may also impact on their applications. This analysis is presented with an emphasis on gauging how each technology may affect the scope of the other.

### 4.1. Comparative Analysis of the Applications of CRISPR/Cas9 and RNAi as Research Tools

Both RNAi and CRISPR/Cas9 are widely used as gene expression modifying research tools for various scientific discovery purposes. Some of the major applications are discussed below, by highlighting the advantages and disadvantages of each one of these two technologies.

*Mammalian in vitro reverse genetic screens*: Reverse genetic screens were used heavily over the past decade to understand several biological processes. RNAi has revolutionized reverse genetic screens to an unprecedented level, emerging as the most valuable tool for conducting gene-function studies in diverse aspects such as cell signaling, host-pathogen interactions, immune response, and cancer mechanisms, among several others. Recently, CRISPR/Cas9 based human genome wide genetic screens have also been reported, indicating that CRISPR/Cas9 could also be used for this purpose [51,52,53,54]. However, there are several advantages for RNAi over CRISPR/Cas9 as a tool of choice for reverse genetic screens.

Cell based genome wide mammalian genetic screens involving gene expression modification tools can be performed theoretically in two different ways: either by transient introduction of gene expression modification tools (e.g., siRNA, shRNA, CRISPR/Cas9 systems) into cells followed by rapid completion of various phenotypic assays, or by using cell clones already having each one of the genes in the genome individually suppressed stably. In reality, all genetic screens conducted till now in cell culture models were mostly performed by transient suppression of gene expression. Although shRNA offers the ability to create cells with stable suppression of individual genes, genome wide collection of individual gene-silenced cells is currently unavailable for any mammal. Similarly, currently no genome wide collection of CRISPR/Cas9 based gene-deleted cells is available for any mammal. Transient use of gene expression modification tools for genetic screens requires that majority of the cells in the treated population of an experimental set up should show suppression of the targeted gene. Tools of both CRISPR (gRNA/Cas9 carrying plasmids or gRNA as synthetic oligonucleotide along with Cas9 separately through plasmids) and RNAi (siRNA, or shRNA as plasmid or virus) can be introduced into nearly all mammalian cells *in vitro*, by optimizing the delivery of the respective reagents. Therefore both technologies have comparable delivery efficiency in *in vitro* cell culture systems. However, when it comes to their efficiency to alter gene expression, both these technologies may perform differently as they stand today. RNAi has a relatively very high efficiency in suppressing gene expression. Optimally designed RNAi tools offers >70% knockdown of the intended target mRNAs upon delivery into the target cells. The ability of CRISPR/Cas9 to edit mammalian genes varies widely, with some of the studies reporting varying efficiency in the range of 1% to 79% [55,56,57]. While NHEJ mediated repair has a high frequency of occurrence, HDR based repair happens with low efficiency. Recently, efforts to enhance the rate of homology directed repair response have been reported [58,59]. Considering various confounding factors such as differences in delivery efficiency, and the probability of all alleles of a gene in a given cell being deleted simultaneously, the total efficiency of CRISPR/Cas9 mediated gene editing becomes a constrained phenomenon. Such a varying editing efficiency mandates that CRISPR/Cas9 tool treated cells should be selected for gene modification before it could be used for genetic screens. However, the relatively high efficiency of RNAi to attenuate transcript levels in each one of the cells that received the appropriate RNAi tool (siRNA or shRNA) makes its effect rather uniform across all cells in an experimental set up. In this regard, at present, RNAi is has some advantage over CRISPR for conducting genetic screens. Another advantage of RNAi is that it can be adopted for genetic screening in both immortalized cell lines, and short lived primary cells that often do not live longer than a week *ex vivo*. The use of varying efficiency CRISPR/Cas9 systems for conducting genetic screens in short lived primary cells will be less feasible because there may not be enough time for selecting gene edited population and performing phenotypic assays. Yet another major limitation of CRISPR/Cas9 system is its inability to simultaneously target all alleles of a given gene in the targeted cells. Because CRISPR/Cas9 targets the DNA element, in order for it to completely modify the expression of a gene, all alleles of the genes may have to be modified. Most of the widely used immortalized human cell lines are aneuploid with triploid or greater chromosome complements, making gene deletion process complex. Contrary to this, because RNAi targets transcripts, it can cause efficient and uniform gene repression. However, generation of validated collection of CRISPRi tools targeting the entire genome of mammals of interest may change this technological imbalance between RNAi and CRISPR for genetic screen applications [35,36,37,60]. Improved algorithms for CRISPRi design have been developed recently, further enhancing the potential of this application [35]. It remains to be established how successful and universally applicable will be the CRISPRi based approach for knocking down the expression of large sets of genes. Whether all genes can be easily suppressed through CRISPRi, or whether different genes might need tedious scanning with large numbers of gRNA to find the one that effectively suppresses gene expression needs to be determined. In this background, it is reasonable to predict that RNAi will most likely remain as the preferred tool for mammalian cell based reverse genetic screens. It is anticipated that newer discoveries will enable enrichment of the on-target specificity of RNAi, enhancing its reliability.

*Transient in vitro gene expression suppression*: There could be instances in which only transient perturbation of gene expression is possible or preferred. For example, essential genes could not be knocked out using CRISPR/Cas9 as this will lead to cell death. In such scenarios, RNAi based transient knockdown of gene transcripts will be the technique of choice for gene expression manipulation. In another set of contexts, one may only need transient alteration of gene expression to deal with or investigate a transient phenomenon. Examples include cellular response to external factors such as infection by pathogens, and drug treatment. Most known pharmacological drugs exert their effects by transiently interacting with the intended cellular target, often proteins. If one has to genetically mimic the effect of transient exposure of cells to a drug, RNAi will be the ideal choice because conventional CRISPR/Cas9 imparts stable genetic change. However, early studies have demonstrated that CRISPRi could also be adapted for transient gene knockdown, without permanently modifying DNA.

*Introduction or correction of mutations*: Studies on the role of genes in cell physiology and cell-environment interactions often needs engineering precise mutations or insertion of DNA segments (e.g., reporter gene) in specific parts of the genes in the endogenous context, as opposed to their complete deletion or repression. Examples include introduction of inactivating point mutations of catalytic activity, deletion of protein domains and so forth. Conversely, there could be situations in which one may want to reverse a mutation in experimental systems. Only the CRISPR/Cas9 system, which targets the DNA, has the ability to introduce or correct mutations into genes. RNAi cannot be used for such precision genome engineering applications because it functions primarily as transcript degradation machinery.

*Enhancement of gene expression*: While both CRISPR/Cas9 and RNAi can down-regulate the expression of genes (respectively irreversibly and transiently), there may arise conditions in which one may want to selectively upregulate the expression of a gene permanently or transiently. RNAi is incapable of directly upregulating the expression of genes because it is a post-transcriptional suppressor of transcript availability. However, CRISPR/Cas9 could be adapted to upregulate the expression of genes. In one approach, CRISPR/Cas9 could be used to stably introduce constitutively active promoter elements to a gene to enhance its expression levels. Alternatively, dCas9 fused to a transcription activator protein by various means (e.g., VP64 protein) could be targeted to any gene of interest using appropriate gRNA, to drive gene expression at an enhanced level transiently (called CRISPR activation, CRISPRa) [35,60,61]. However, the ease and success rate of CRISPRa is yet to be evaluated by testing against large sets of genes. Thus, CRISPR but not RNAi offers the ability to enhance gene expression.

### 4.2. Comparative Analysis of the Clinical Applications of CRISPR/Cas9 and RNAi

Gene modifying technologies hold the promise of therapeutic applications to treat both heritable genetic as well as non-genetic diseases such as infectious diseases [62,63,64]. Accordingly, both RNAi and CRISPR are predicted to have several human therapeutic applications. We selectively present here some of the major core concepts by which RNAi and CRISPR could potentially be used for clinical applications, along with a discussion on how each technology will affect the acceptance of the other.

*In vivo* gene therapy has always been viewed as a very promising approach for curing many monogenic, complex genetic and non-genetic medical conditions. Gene therapy tools could be either directly used *in vivo*, or used *ex vivo* to generate gene corrected cells for adoptive therapeutic transfer [65]. The curative effects of gene therapy are often achieved by introducing either a desired gene, correction of a mutation, ablation of the expression of a gene containing gain of function mutation, or by activating a compensatory gene, into the tissues of interest, depending on the context. Introduction of genes and correction of point mutations in target host cell genomes can be best achieved though HDR. Ablation of gene expression can be achieved by NHEJ. Examples for diseases that would benefit from therapeutic correction of mutation include gene therapy for severe combined immunodeficiency and cystic fibrosis [66,67]. An example for curative activation of a gene other than the disease causing mutation-harboring gene is the selective upregulation of γ-globin for treating sickle cell anemia arising from mutation in β-thalassemia gene [68].

Many of the genetic diseases will benefit from gene therapy through stable gene editing. As RNAi is unable to either stably introduce gene segments/mutations or induce activation of genes, CRISPR/Cas9 system will be the method of choice for such gene therapy applications. Because CRISPR based gene editing is heritable, technically, one will have to introduce the genomic changes into host cells only a single time, and the physiological effect might last as long as the targeted cells are viable, or will be inherited to daughter cells in the case of dividing cells. In contrast to CRISPR/Cas9, *in vivo* application of RNAi is limited only to the instances in which the expression of genes has to be suppressed post-transcriptionally. RNAi can be made stable and heritable only by using viral delivery platforms such as a lentiviral platform which can integrate into host genome. Because lentiviral integration into mammalian cells *in vivo* can give rise to undesirable medical outcomes in the long term, this approach may be less desirable [69].

Successful *in vivo* use of CRISPR/Cas9 requires an adequate delivery platform, coupled with efficient DNA repair response. Due to their potential tumorigenic properties, retroviruses and lentiviruses may not be the ideal choice for direct *in vivo* delivery of CRISPR/Cas9 systems. Alternative delivery platforms include those based on adenovirus and adeno-associated virus. However, the large size of Cas9 protein is often a limitation for the *in vivo* delivery of CRISPR/Cas9 systems using some of the well-established vectors such as those based on adenovirus. Recently, adeno-associated viruses have been reported as efficient vector systems for *in vivo* gene editing using a smaller variant of Cas9 [3]. Similarly, *in vivo* delivery of RNAi has also been achieved using adeno-associated virus vectors [70]. However, unlike as in CRISPR/Cas9 systems, the size of the targeting insert that needed to be carried in the delivery vector is very small for RNAi.

Another important aspect impacting on the applications of various gene editing technologies in gene therapy *in vivo* is the host immune response to both the delivery vehicle and editing associated proteins. Many times, a host mounts immune response to delivery vehicles such as adenoviruses and adeno-associated viruses. Similarly, Cas9 itself may be immunogenic. For example, a recent study that used adenovirus mediated delivery of CRISPR/Cas9 to target the *Pten* gene in mouse liver to treat nonalcoholic steatohepatitis observed that the murine host developed adaptive immune response to both the vector and the Cas9 protein [71]. On the other hand, in the case of RNAi, only the immune response to vector is prevalent during *in vivo* delivery using viral delivery platforms.

The efficiency of DNA repair mechanisms activated by Cas9 mediated DNA break will also impact its *in vivo* applications. Due to its inherently slow rate, HDR mediated gene editing might yield only fewer desired editing events in the target tissues when applied *in vivo*. However, if the gene edited cells have any selective advantage over the unedited disease cells, even a small starting population of gene edited cells might be enough to eventually display a curative effect.

There are many human diseases that could be treated by taking cells, including stem cells, from the patient and correcting the mutations *ex vivo* before re-introducing cells back into the patients [65,66,67,72]. Examples for this include therapeutic use of CRISPR/Cas9 mediated *ex vivo* gene edited cells to treat Duchenne muscular dystrophy and cystic fibrosis [73,74]. Such applications will require the generation of cells with stable genetic changes. Similarly, stem cells or induced pluripotent stem cells could be engineered to have heritable curative genetic modifications for therapeutic purposes [66]. CRISPR would be the ideal choice to create such stable genetic changes. Another similar growing area of medical research that will benefit from genome engineering involves the generation of chimeric antigen receptor T cells (CART), to train T cells to detect specific epitopes [75,76]. The CRISPR/Cas system will be ideal for generating CART cells. None of these therapeutic applications can be achieved through RNAi. CRISPR could in fact be used to edit genes without having the need to leave the delivery vehicle (e.g., potentially dangerous lentivirus vector) in the target cells indefinitely, because the genetic change introduced by Cas9 through NHEJ/HDR is stable and irreversible. For example, a gRNA can be delivered as synthesized oligonucleotides and Cas9 could be delivered as purified protein [77,78]. This evidence points to CRISPR as a supreme technology over RNAi for gene therapy using *ex vivo* gene edited cells.

On the other hand, there may also arise specific instances in which transient depletion of a mammalian gene *in vivo* is needed for therapeutic purpose. For example, transient attenuation of expression of an essential pro-pathogen host factor may help to clear an acutely infecting pathogen, or brief attenuation of the expression of a host gene may alleviate an acute clinical condition. Because these or similar applications only need transient repression of the expression of host gene to treat the disease, both RNAi and CRISPRi could be used alike, both for direct *in vivo* modification and adoptive transfer of *ex vivo* gene edited cells. However, while RNAi is proven to work efficiently in most mammalian cell types if efficient delivery can be achieved, whether CRISPRi would work in specific cell subtypes *in vivo* is yet to be determined. Whether chromosomal topology or prior formation of regulatory protein complexes at a particular genomic location *in vivo* affects the ability of CRISPRi to repress transcription is currently not well defined. Moreover, RNAi could be delivered *in vivo* through the delivery of siRNAs. In this regard, RNAi may offer advantages over CRISPRi for such transient *in vivo* gene expression suppression purposes.

*Targeting viral pathogens*: There are several viruses that infect humans with severe consequences. In principle, gene editing technologies could be used to directly edit the genomic DNA of viruses such as hepatitis B virus, resulting in the elimination of the virus [79]. Similarly, HIV could also be targeted by mutating its genetic elements integrated into the host genome. Such applications could only be achieved through CRISPR, and not through RNAi because RNAi will only target the viral transcripts. Besides DNA viruses, there are also several RNA viral pathogens infecting humans. The ability of CRISPR to edit RNA is still not fully understood and widely exploited. However, there were recent reports attempting to edit RNAs, including that of an RNA virus, using CRISPR, hinting that eventually RNA targeting applications of CRISPR may become more efficient [80]. These studies used multicomponent CRISPR systems, unlike the popularly used simple Cas9 based systems, making their clinical use a major challenge. As the technologies stand today, adaptation of CRISPR systems as a versatile tool to edit RNA is yet to be established. Contrary to this, RNAi has been widely used for directly targeting the RNA of RNA viruses [81,82]. In conclusion, while CRISPR offers enormous potential to directly inactivate DNA viral pathogens, RNAi currently stands as the most promising tool to target RNA viruses.

### 4.3. Technical Considerations Impacting the Applications of CRISPR/Cas9 and RNAi

There are several technical and methodological aspects of CRISPR/Cas9 and RNAi that may also contribute to the choice of applications that each one of them could be associated with. Some of these are listed below.

*Time*: The time needed for each one of these techniques to manifest the intended effect on gene structure or expression varies greatly, and this may profoundly affect the choice for specific applications. RNAi exhibits rapid effects on gene expression. In around 24 h, RNAi treatment can result in significant attenuation of gene expression repression uniformly in the majority of the treated cells. However, the low to moderate efficiency of CRISPR requires selection of cells having desired InDels in all alleles, and this process may take up to a month or more depending on the specific needs. However, CRISPRi and CRISPRa may alter gene expression relatively faster, by 2–5 days [36].

*Nature of the phenotype*: CRISPR induces a absolute null phenotype that is irreversible [20]. However, RNAi, CRISPRi and CRISPRa impart reversible and incomplete modification of gene expression [35,37,40,60].

*Genetic information needed for designing targeting systems*: Design of RNAi tools need information on the sequence of the corresponding gene transcript, and may not require substantial information of the sequence of the corresponding gene. On the contrary, CRISPR requires the gene sequence information [4,20]. Furthermore, CRISPRi and CRISPRa design requires proper information on the transcription start site of the target gene [35,37,40,60].

*Target specificity*: It is widely established that RNAi has off-target effects [83]. Although newer design principles have greatly contributed to reduce off-target effects, there still exists no methodology to completely eliminate RNAi induced off targets [33]. Currently, the off-target effect generation potential of CRISPR/Cas9 system in mammalian cells is not yet fully appreciated. However, there are several recent reports that identified noticeable off-target potential of CRISPR technology [84,85,86,87]. Scientists have also modified CRISPR/Cas9 methodology to minimize its potential off-target effects [88]. Delivery of Cas9 as mRNA, and gRNA-Cas9 as ribonucleoprotein complex, have been proposed to reduce off-target due to the short cellular life of these formulation, as opposed to plasmid based delivery [77,78,89]. In addition, a recent study reported the generation of a mutant Cas9 with minimal off-target potential [90]. All these approaches indicate that newer discoveries will eventually reduce the off-target potential of CRISPR/Cas9 systems.

*Targetable genes*: RNAi can be used to target literally any protein-coding gene. The requirement of a short protospacer-adjacent motif at the target gene site makes the application of CRISPR/Cas9 tied to the presence of this sequence [5]. This raises the question of how many human genes could be edited by CRISPR/Cas9. Use of other CRISPR/Cas systems may help to increase the targetable mammalian gene coverage. Recently, Cas9 has been adapted to tolerate variations in protospacer-adjacent motif requirement through *in vitro* evolution [91]. RNAi cannot be used to edit genetic elements such as non-transcribed noncoding regulatory regions. Unlike RNAi, CRISPR/Cas9 can be used to target non-transcribed genomic segments [92]. Another factor that affects the efficacy of the gene modulation by RNAi and CRISPR/Cas9 is the state of the cell. While in theory, RNAi can target transcripts in any phase of the cell cycle, host response to CRISPR mediated DNA damage has dependence on the cell cycle phase. While NHEJ can operate in all phases of cell cycle, HDR based DNA repair system happens predominantly during S/G2 phase, limiting their applications in genome editing [93]. In such a scenario, NHEJ can dominate over HDR dependent repair, and may yield potentially unintended results.

## 5. Conclusions

As extensively described in this analysis, both CRISPR/Cas9 and RNAi are powerful tools for gene manipulations. CRISPR/Cas9 is certainly more versatile and superior to RNAi as it can be used to induce InDels, both repress or activate gene expression, and cause both heritable and non-heritable genomic changes. In addition, the emergence of newer gene editing tools such as the Cpf1 enzyme may eventually strengthen the portfolio of applications that can be achieved by CRISPR mediated genome engineering [94]. These qualitative advantages of CRISPR/Cas9 poise it to effectively dominate RNAi in diverse genetic applications in both clinical and research tool applications fields. However, based on the analysis of the currently available facts, it is reasonable to conclude that RNAi will also have a certain unique space in both biomedical research and clinical applications, at least in the near future. The biggest threat to RNAi is from CRISPRi. However, wider application of CRISPRi in the mammalian system is yet to be established through extensive experimental validations at a genome scale. Therefore, it is reasonable to predict that the fate of RNAi will greatly depend on the success of CRISPRi. One of the biggest technical applications of RNAi that might be unaffected by CRISPR in the short term future will be in the field of arrayed reverse genetic screens. Another area where RNAi may find its own niche will be for targeting RNA-viral pathogens. In conclusion, while CRISPR/Cas9 will predictably dominate applications of gene modifying technologies in the long term, RNAi will likely continue to survive with restricted domains of applications.
